# First Serological Evidence of Avian Metapneumovirus and Infectious Laryngotracheitis Virus in Commercial Poultry in the Ashanti Region of Ghana

**DOI:** 10.1002/vms3.70971

**Published:** 2026-04-28

**Authors:** Patrick Mensah Amponsah, Kwadwo Boampong, Augustina Angelina Sylverken

**Affiliations:** ^1^ Department of Theoretical and Applied Biology Kwame Nkrumah University of Science and Technology (KNUST) Kumasi Ghana; ^2^ Kumasi Veterinary Laboratory Amakom Ghana; ^3^ Kumasi Centre for Collaborative Research in Tropical Medicine Kwame Nkrumah University of Science and Technology (KNUST) Kumasi Ghana

**Keywords:** avian, avian metapneumovirus, infectious laryngotracheitis, respiratory diseases, Ghana

## Abstract

**Background:**

Avian metapneumovirus (aMPV) and infectious laryngotracheitis virus (ILTV) are globally important respiratory pathogens in poultry. However, their serological prevalence has not been established in Ghana. The study therefore sought to serologically detect aMPV and ILTV in commercial poultry in the Ashanti Region of Ghana.

**Methods:**

This cross‐sectional serological study was conducted in the Ashanti Region, one of the main poultry‐producing zones in Ghana. Antibodies to aMPV and ILTV were determined in serum samples collected from 300 clinically healthy chickens from thirteen large commercial farms, using commercially available indirect ELISA kits (IDvet, France).

**Results:**

Overall seroprevalence was 49.0% (147/300; 95% CI: 43.3%–54.7%) for aMPV and 7.0% (21/300; 95% CI: 4.4%–10.6%) for ILTV. Farm‐level seroprevalence of aMPV ranged from 0% to 100%, while ILTV ranged from 0% to 40%. Co‐exposure with both viruses was detected in 5.0% (15/300) of birds. Fisher's exact test revealed a statistically significant positive association between aMPV and ILTV seropositivity (*p* = 0.041; odds ratio = 2.78; 95% CI: 0.98–8.99).

**Conclusion:**

The study provides the first serological findings of exposure of aMPV and ILTV in commercial poultry flocks in Ghana. The high levels of aMPV together with the ILTV detection are a true indication that the need to improve respiratory disease surveillance, diagnostic capacity and analyse potential vaccination options of the Ghanaian poultry industry is urgent.

## Graphical abstract

This study reports the first serological evidence of avian metapneumovirus and infectious laryngotracheitis virus in commercial poultry in Ghana. High aMPV seroprevalence (49%) and lower ILTV prevalence (7%) were detected, with some coinfection. Findings highlight the urgent need to strengthen respiratory disease surveillance, diagnostics and vaccination strategies in the poultry industry.

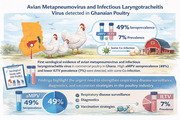

**Abbreviations**
aMPV: avian metapneumovirus
ELISA: enzyme‐linked immunosorbent assay
IBV: infectious bronchitis virus
ILTV: infectious laryngotracheitis virus
KCCR: Kumasi Centre for Collaborative Research
KNUST: Kwame Nkrumah University of Science and Technology
NDV: Newcastle disease virus
OD: optical density
S/P: sample‐to‐positive ratio
VSD: Veterinary Services Department

## 1 Introduction

High morbidity, mortality and reduced productivity in poultry production caused by infectious diseases impact greatly on the poultry industry (Mensah et al. 2023). These are particularly pronounced in low resource settings where there is poor farm biosecurity (Ouma et al. 2023). Over the past years, all major disease outbreaks in the poultry industry in Ghana have involved respiratory pathogens (Mensah et al. 2023; Albert et al. 2024; Appiah et al. 2025). From outbreaks of highly pathogenic avian influenza (HPAI) to coinfections of low pathogenic avian influenza (LPAI) with infectious bronchitis (IB), along with the endemic presence of Newcastle disease (NCD) and Mycoplasmosis, respiratory pathogens continue to place a heavy financial burden on the poultry industry though the majority of them have not been established as zoonotic pathogens except for avian influenza. In Ghana alone, GHS 44 million (approximately $440,000) was spent in 2022 just to control avian influenza (Albert et al. 2024; Asante et al. 2016; Burn et al. 2022).

Contributing to the high susceptibility of avian to respiratory infections is the unique anatomical features of their respiratory system. Their complete tracheal rings and reduced mucociliary clearance enhances the persistence of pollutants and pathogens in the respiratory system leading to diseases (Burn et al. 2022). This predisposition is compounded by environmental stressors and the frequent occurrence of coinfections under field condition (Liu et al. 2025).

In low‐income countries such as Ghana, poultry diseases are often diagnosed mainly through the examination of gross pathological lesions, because advanced laboratory methods like molecular and serological diagnostic methodologies with their higher specificity and sensitivity are not widely available (Ofori et al. 2024; Enyetornye et al. 2025). This constraint makes it difficult to differentiate pathogens with overlapping clinical signs, increasing the likelihood of misdiagnosis, underreporting and failure to detect emerging or neglected pathogens such as ILTV and aMPV (Enyetornye et al. 2025).

Although clinical presentations suggestive of these infections have been observed, Ghana currently lacks any published serological evidence of exposure to ILTV or aMPV in poultry. In contrast, seroepidemiological investigations globally have established the widespread circulation of both viruses, often in the absence of overt clinical disease (Adetolase et al. 2020; Salles et al. 2023; Bakre et al. 2025; Balcha et al. 2023; Abebe et al. 2024). Studies across Europe, Asia and South America have demonstrated varied seroprevalence levels using enzyme‐linked immunosorbent assays (ELISA) confirming both viruses as significant contributors to respiratory disease complexes in poultry (Gallian et al. 2017; Roy et al. 2015; Gowthaman et al. 2020). In Africa, limited serological studies from Nigeria, Egypt and South Africa have reported exposure in domestic poultry (Adetolase et al. 2020; Bakre et al. 2025; Balcha et al. 2023; Nagy et al. 2018). The absence of such data in Ghana is not indicative of absence of infection but rather reflects surveillance gaps that obscure the true epidemiological landscape.

It is worthy of mention that both ILTV and aMPV possess distinct pathophysiological mechanisms that contribute to under recognition in field conditions. ILTV, an alpha herpesvirus of the Herpesviridae family, exhibits epithelial tropism for the tracheal, laryngeal and conjunctival mucosa (Munuswamy et al. 2019). This leads to focal necrosis, haemorrhagic inflammation and sloughing of the respiratory epithelium. Clinically, infections manifest as gasping, conjunctivitis and dyspnoea, and in severe cases, tracheal obstruction (Balfour‐Lynn and Wright 2019). Avian metapneumovirus (aMPV), a pneumovirus of the Paramyxoviridae family, primarily targets the upper respiratory tract, inducing ciliostasis, epithelial damage and impaired mucociliary clearance. This predisposes infected birds to secondary bacterial infections, often resulting in more severe disease clinical presentation including the swollen head syndrome (Salles et al. 2023; Goraichuk et al. 2024). Both viruses are capable of causing subclinical or mild diseases, especially in partially immune populations, and exhibit clinical overlap with endemic pathogens such as NDV and IBV, further complicating field diagnosis (Bello et al. 2018).

These biological characteristics, combined with infrastructural diagnostic constraints, underscore the urgent need for serological surveillance as a tool to uncover exposure and clarify pathogen circulation in Ghanaian poultry. This study addresses a critical knowledge gap by providing the first serological evidence of exposure to aMPV and ILTV both of which have been associated with marked production losses and increased mortality (Ofori et al. 2024; Munuswamy et al. 2019), thereby laying the foundation for future molecular confirmation, targeted vaccination strategies and integrated respiratory disease control.

## 2 Materials and Methods

### 2.1 Study Area

**FIGURE 1:**
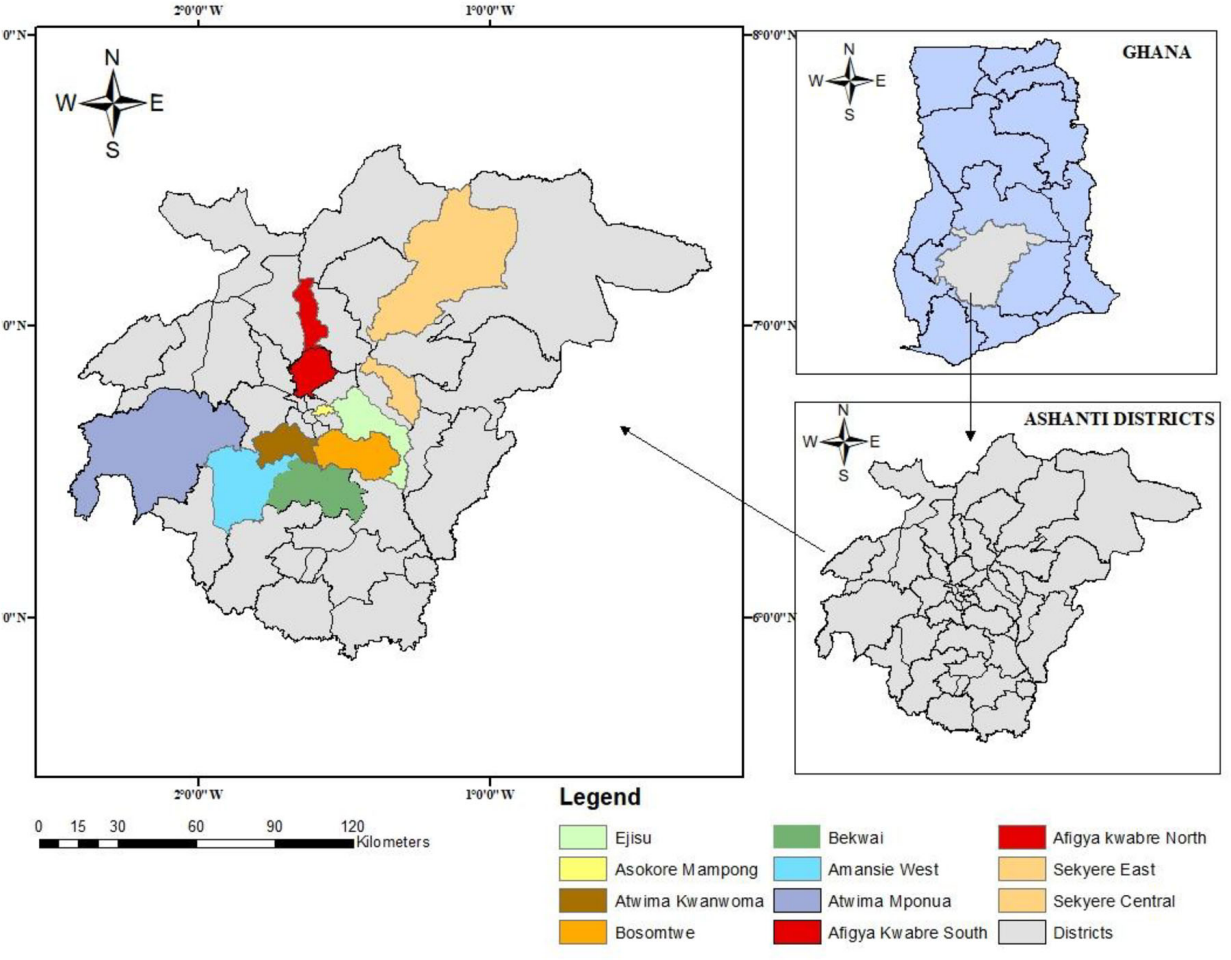
Map of Ashanti Region highlighting districts where samples were collected.

The study was conducted in the Ashanti Region of Ghana, a major hub of commercial poultry production characterized by a high concentration of small‐ to medium‐scale farms, particularly in urban and peri‐urban districts. Poultry production in the region is predominantly intensive, with layer birds raised under deep litter or battery cage systems to meet the growing demand for eggs and poultry products. However, the sector is often challenged by issues such as high stocking densities, variable biosecurity practices and frequent movement of birds and farm inputs, which can facilitate the spread of infectious diseases. Thirteen farms were randomly selected from different districts within the region. From each of the selected farm, layer chickens were randomly selected and sampled (Figure 1).

### 2.2 Study Design, Sampling Technique and Sample Size

This cross‐sectional study was conducted between June and November 2022 in the Ashanti Region of Ghana.

Thirteen districts out of the 43 districts in Ashanti Region were randomly selected. Within each selected district, a large commercial poultry farm was randomly selected.

The sample size was calculated using the formula for prevalence studies:

$$n=\frac{Z^{2}p\left(1-p\right)}{d^{2}}\;\;\times \mathrm{DE}$$

A prevalence of 21.2% for aMPV (Munuswamy et al. 2019) was used as the basis for the calculation of the sample size for the study. With a precision (*d*) of 5%, and *Z* at a 95% confidence interval (CI) (1.96), and a design effect (DE) of 1.17 to account for clustering. A total of 300‐layer birds were sampled.

### 2.3 Inclusion and Exclusion Criteria

All clinically healthy chickens from the selected commercial farms in the Ashanti Region were eligible for inclusion. Birds exhibiting signs of severe illness, injury or debilitation were excluded to avoid compromising animal welfare and data quality. Furthermore, birds younger than 2 weeks of age were excluded due to the potential confounding effects of maternal antibodies and immune system immaturity on serological outcomes. Birds selected were not vaccinated as at the time of sample taking, and they were egg producers.

### 2.4 Sample Collection and Serological Analysis

Whole blood was collected using a 2 mL syringe. Sera were subsequently harvested and immediately stored at −20°C until serological analysis.

Serum samples were tested for IgG using two commercial ELISA kits from IDvet (Grabels, France): the ID Screen Infectious Laryngotracheitis Virus Indirect ELISA (ILTS ver. 1121) and the ID Screen Avian Metapneumovirus Indirect ELISA (MPVS ver. 0416).

The tests were done according to the manufacturer's instructions.

The optical density (OD) of each sample was measured at 450 nm using a BioTek ELx800 microplate reader (Agilent Technologies, USA). Each test plate was considered valid because the positive and negative controls met the manufacturer's requirements, where the mean OD of positive controls was at least 0.250 and at least three times higher than that of the negative controls. For every sample, the sample‐to‐positive (S/P) ratio was then calculated using the formula provided by the manufacturer.

$$\frac{S}{P}\%=\frac{OD_{\mathrm{sample}}\;-\;OD_{\mathrm{negative}}}{OD_{\mathrm{positve}}-\;OD_{\mathrm{negative}}}\times 100$$

The antibody titre was derived using the log‐transformed equations recommended by IDvet.

For infectious laryngotracheitis (ILT), the formula used was:

$$\mathrm{lo}g_{10}\left(\mathrm{titre}\right)=1.10\times \mathrm{lo}g_{10}\left(S/P\right)+3.361$$

For aMPV, the corresponding equation was:

$$\mathrm{lo}g_{10}\left(\mathrm{titre}\right)=1.09\times \mathrm{lo}g_{10}\left(S/P\right)+3.360$$

The actual titre value was then obtained as:

$$\mathrm{titre}\;=10\times \mathrm{lo}g_{10}\;\left(\mathrm{titre}\right)$$

For the ID Screen ILT Indirect test, samples with S/P≤0.5 (titre ≤ 811) were considered negative, while those with $\frac{S}{P}>0.5$ (titre ≥ 811) were positive. For the ID Screen aMPV Indirect test, samples with S/P≤0.2 (titer ≤ 396) were considered negative, and those with S/P>0.2 (titre ≥ 396) were positive.

### 2.5 Data Analysis

Data were analysed using R version 4.5.1. Descriptive statistics were used to calculate the number and percentage of samples positive for each virus. The prevalence (%) was determined by dividing the number of positive samples by the total number tested and multiplying by 100. The 95% CIs for prevalence estimates were computed using the binomial exact method. To assess the relationship between aMPV and ILTV infections, a Fisher's exact test was performed on a 2 × 2 contingency table of infection status because two out of the four cells (50%) had expected counts less than 5, exceeding the 25% threshold and violating the chi‐square test assumption of adequate expected frequencies. As Fisher's exact test assumes fixed marginal totals and does not rely on large‐sample approximations, it provides a more accurate and valid estimate of association under these conditions. The test provided the odds ratio (OR), 95% CI and *p*‐value to evaluate the strength and significance of the association. A *p*‐value less than 0.05 was considered statistically significant.

### 2.6 Ethical Consideration

This study was conducted in accordance with the ethical standards and guidelines of the Veterinary Services Department (VSD), Ghana. Ethical approval for the research was obtained from the VSD Research Ethics Committee under the reference number VSD/RES GEN/05/22. All procedures involving animals were carried out following established animal welfare regulations and biosecurity protocols to minimize distress and ensure humane treatment.

## 3 Results

### 3.1 Overall Prevalence of aMPV and ILTV

In this study, a total of 300 samples from 13 farms were tested for antibodies against ILTV and aMPV using ELISA technique. Out of 300 serum samples, 21 (7%) tested positive for ILTV and 147 (49%) for aMPV.

### 3.2 Sample Distribution Across District

Table 1 presents the number of serum samples collected from each district included in the study.

**TABLE 1:** Number of serum samples collected from each of the district for serological analysis.

District	Number of samples
Afigya‐Kwabre South	27
Amansie Central	36
Asokore‐Mampong	33
Atwima‐Kwanwoma	12
Atwima‐Mponua	21
Atwima‐Nwabiagya North	12
Bekwai	24
Bosomtwe	24
Ejisu	9
Kumasi	24
Sekyere Central	30
Sekyere East	48

### 3.3 District Prevalence of aMPV and ILTV

**FIGURE 2:**
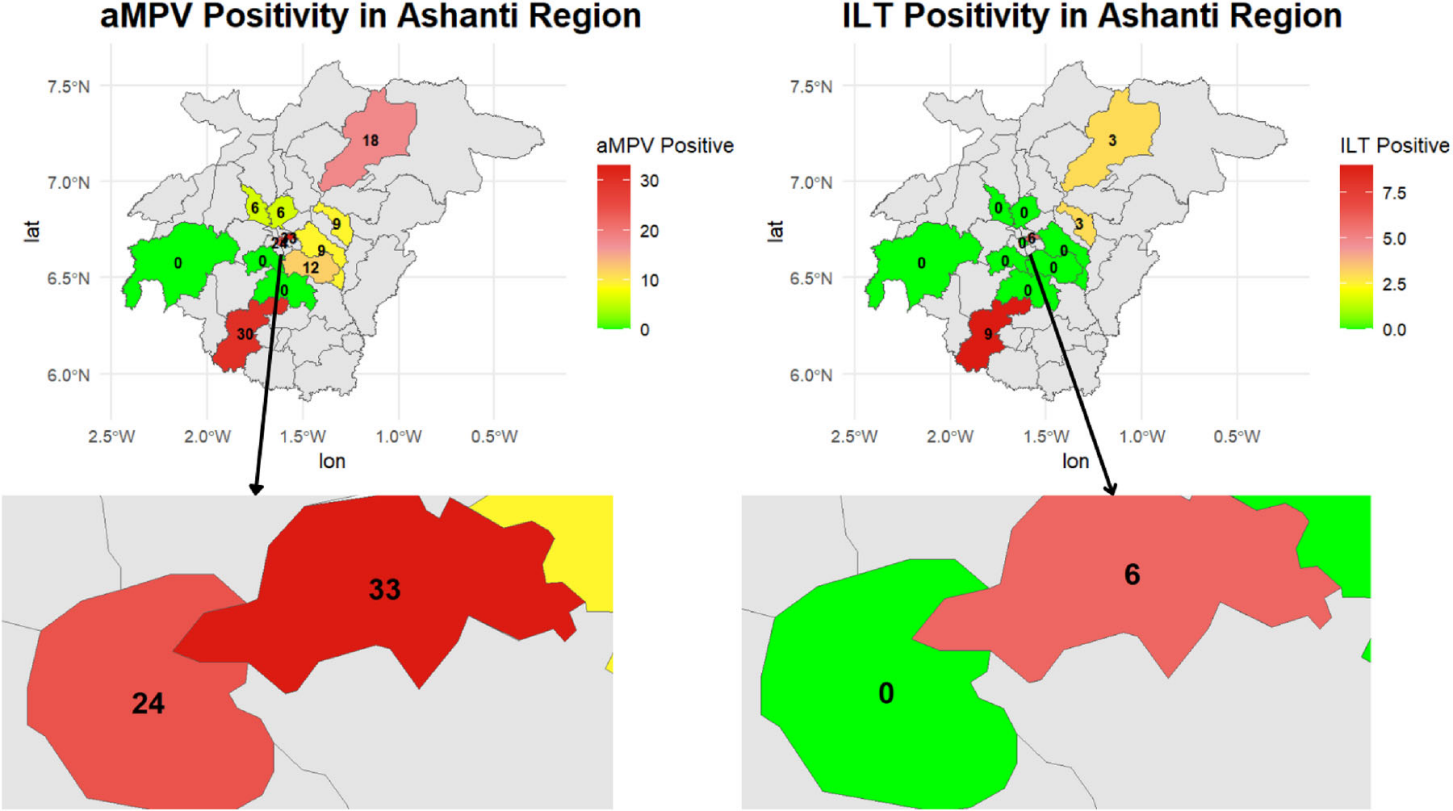
Comparisom of aMPV and ILT seroprevalence across districts.

The prevalence was highest for aMPV, with some districts (Ejisu, Kumasi and Asokore‐Mampong) showing up to 100% positivity, while ILTV detection was generally lower and more varied across farms (Figure 2).

### 3.4 Co‐Exposure of aMPV and ILTV

Of the 300 samples tested for both aMPV and ILT, 15 (5%) were positive for both viruses, 132 (44%) were positive for aMPV only, 6 (2%) were positive for ILT only and 147 (49%) were negative for both (Table 2).

**TABLE 2:** Co‐exposure prevalence of aMPV and ILTV among 300 poultry serum samples.

	ILT positive	ILT negative	Total
aMPV positive	15	132	147
aMPV negative	6	147	153
Total	21	279	300

### 3.5 Fisher's Exact Test of Association Between aMPV and ILTV

Fisher's exact test showed a significant association between aMPV and ILTV infections (*p* = 0.041, OR = 2.78, 95% CI: 0.98–8.99), indicating that aMPV‐positive samples were about 2.8 times more likely to be ILTV‐positive.

## 4 Discussion

This study provides the first serological evidence of exposure to aMPV and infectious laryngotracheitis virus (ILTV) among commercial poultry in the Ashanti Region of Ghana. The results show that 49% of the sampled chickens were positive for aMPV antibodies, and 7% were positive for ILTV antibodies. These findings indicate that both viruses are circulating among poultry flocks in the region, even though there have been no previous official reports of these infections in Ghana.

The high seroprevalence of aMPV found in this study is consistent with findings from other African countries such as Nigeria, Ethiopia and Egypt, where similar levels of exposure have been reported (Adetolase et al. 2020; Bakre et al. 2025; Nagy et al. 2018; Habte et al. 2022). aMPV is known to spread easily in densely populated farms and can persist in flocks without clear clinical signs, especially when birds have partial immunity or when infections are mild (Goraichuk et al. 2024; Suarez et al. 2020). The high detection rate suggests that the virus might be widely distributed across farms in Ghana and could be contributing to respiratory disease problems that are often attributed only to more commonly known pathogens such as Newcastle disease virus (NDV) and infectious bronchitis virus (IBV) (Ayim‐Akonor et al. 2018). In contrast, ILTV seroprevalence was relatively low (7%) but still important. This is not surprising since ILTV is known to cause sporadic outbreaks and may remain undetected in farms lacking molecular diagnostic capacity (Gowthaman et al. 2020). The detection of ILTV antibodies in apparently healthy birds indicates that the virus may be circulating silently, possibly through recovered carriers or subclinical infections. This supports the need for molecular confirmation and continuous surveillance. The serological detection of aMPV and ILT in intensively reared commercial poultry in Ghana reveals substantial husbandry and biosecurity gaps (Adomako et al. 2024). In Ghana presently, no vaccination programs for these pathogens exist, hence, seropositivity unequivocally indicates natural exposure rather than vaccine‐induced antibodies (Enyetornye et al. 2025). Avian respiratory viruses such as aMPV and ILTV are highly contagious and can be introduced into flocks via contaminated equipment, personnel or fomites especially in settings with insufficient farm access controls and disinfection routines (Gowthaman et al. 2020; Munuswamy et al. 2019; Mo and Mo 2025). For instance, ILTV can persist in dust, litter and on farm machinery, maintaining infectivity during prolonged intervals if not properly neutralized (Roy et al. 2015). Detection of antibodies to these two important viral respiratory pathogens of poultry in the Ashanti Region warrants expansion of this study to other heavy poultry production zones of Ghana. These findings also suggest the need for diagnostic laboratories in Ghana to include aMPV and ILT in screening panels for poultry viral respiratory pathogens. This study also provides a basis to start a national dialogue on possible revision of Ghana's national poultry vaccination schedule and the need for enhanced surveillance for these pathogens.

The serological evidence underscores systemic vulnerabilities across Ghana's poultry value chain. Commercial operations frequently procure chicks, feed and equipment from diverse suppliers, creating multiple potential entry points for pathogens when biosecurity measures are inadequate (Adomako et al. 2024; Enyetornye et al. 2025). Empirical studies have identified poor farm‐level biosecurity particularly the failure to quarantine newly introduced stock as a critical risk factor for the incursion of respiratory pathogens such as ILTV (Balcha et al. 2023). Furthermore, the absence of structured disease surveillance and limited farmer awareness facilitates the silent circulation of agents like aMPV and ILTV, increasing the likelihood of endemic establishment and amplifying the risk of future outbreaks (Etuah et al. 2020; Samy and Naguib 2018).

Samples for this study were taken from birds raised either in deep litter or battery cage systems. Although the findings were not stratified into these two categories‐ deep litter or battery cage, it is important to note how these two systems may modulate exposure pressure and transmission pathways for these two viruses. Deep litter environments have been identified to accumulate organic material and aerosols where respiratory secretions and viral particles can persist, increasing the likelihood of repeated mucosal exposure and subclinical seroconversion events (Hostyn et al. 2025). In high‐density floor systems, frequent bird–bird contact and shared airspace amplify horizontal spread of respiratory pathogens, and the litter itself acts as a fomite reservoir when drying, turning or removal is suboptimal (Hostyn et al. 2025). ILT and aMPV epidemiology consistently associate intensive, high‐density housing and imperfect environmental hygiene with elevated transmission risk, which plausibly translates into higher seropositivity in deep‐litter commercial flocks compared with caged systems that physically separate birds from excreta and reduce litter‐mediated contact (Mo and Mo 2025; Liu et al. 2025; Grace et al. 2024). Field studies from African commercial operations further support this pattern. For instance, recent ILTV surveys in Ethiopia show substantial seroprevalence in commercial flocks, with risk factors linked to management practices that are typical of floor‐based systems (Abebe et al. 2024).

At the farm level, the prevalence of aMPV varied widely across the 13 farms. Ten farms recorded aMPV antibodies, with prevalence levels ranging from 22% to 100%, while three farms had no detectable antibodies. In contrast, ILTV antibodies were detected in only four farms, with prevalence ranging between 10% and 25%. Some farms showed very high aMPV positivity, suggesting ongoing or recent exposure, whereas most farms had little to no ILTV detection.

The significant association found between aMPV and ILTV infections suggests that coinfection may occur in some farms. This relationship could worsen clinical symptoms and production losses, as both viruses damage the respiratory tract and make birds more susceptible to secondary bacterial infections. Similar coinfection patterns have been reported in other countries, where birds exposed to multiple respiratory viruses often experience more severe disease outcomes (Samy and Naguib 2018).

The findings of this study highlight key gaps in Ghana's poultry health surveillance system. Many farms rely on clinical signs alone for disease diagnosis, which makes it easy for these viruses to go unnoticed. The lack of vaccination and limited diagnostic testing capacity could allow the continuous spread of these pathogens. Therefore, the detection of antibodies against aMPV and ILTV calls for strengthened surveillance, improved biosecurity and consideration of vaccination strategies, especially for large commercial farms.

## 5 Conclusion

This study confirms for the first time that aMPV and ILTV are present among commercial poultry farms in the Ashanti Region of Ghana. The high level of exposure to aMPV and the detection of ILTV, even at lower levels, show that these viruses are circulating silently in the poultry population.

The significant link between aMPV and ILTV infections also points to the possibility of coinfections that could increase disease severity.

These findings emphasize the need for continuous surveillance and improved diagnostic capacity. Future research should include molecular testing and genetic characterization of these viruses to support early detection and the design of effective control measures in Ghana's poultry industry.

## Author Contributions

**Patrick Mensah Amponsah**: conceptualization, investigation, writing – original draft, writing – review and editing, formal analysis, data curation, methodology. **Kwadwo Boampong**: conceptualization, writing – original draft, writing – review and editing, supervision. **Augustina Angelina Sylverken**: conceptualization, writing– original draft, writing – review and editing, methodology, supervision.

## Funding

The authors have nothing to report. The study was self‐funded by the authors, with technical support from the Kumasi Centre for Collaborative Research in Tropical Medicine (KCCR) at the Kwame Nkrumah University of Science and Technology (KNUST) and logistical support from the Kumasi Veterinary Laboratory.

## Ethics Statement

This study was conducted in accordance with the ethical standards and guidelines of the Veterinary Services Department (VSD), Ghana. Ethical approval for the research was obtained from the VSD Research Ethics Committee under the reference number VSD/RES GEN/05/22. All procedures involving animals were carried out following established animal welfare regulations and biosecurity protocols to minimize distress and ensure humane treatment.

## Conflicts of Interest

The authors declare no conflicts of interest.

## Data Availability

All data generated or analysed during this study are included in this study.
